# Melatonin Activates Endoplasmic Reticulum Stress and Apoptosis in Rats with Diethylnitrosamine-Induced Hepatocarcinogenesis

**DOI:** 10.1371/journal.pone.0144517

**Published:** 2015-12-11

**Authors:** Andrea Janz Moreira, Raquel Ordoñez, Carlos Thadeu Cerski, Jaqueline Nascimento Picada, Andrés García-Palomo, Norma Possa Marroni, Jose L. Mauriz, Javier González-Gallego

**Affiliations:** 1 Center of Experimental Research, Hospital de Clínicas de Porto Alegre, Porto Alegre, Brazil; 2 Universidade Federal do Rio Grande do Sul, Porto Alegre, Brazil; 3 Graduate Program in Cell and Molecular Biology Applied to Health, Universidade Luterana do Brasil, Canoas, Brazil; 4 Institute of Biomedicine (IBIOMED), University of León, and Centro de Investigación Biomédica en Red de Enfermedades Hepáticas y Digestivas (CIBERehd), León, Spain; 5 Service of Oncology, Complejo Asistencial Universitario de León, León, Spain; University of Navarra School of Medicine and Center for Applied Medical Research (CIMA), SPAIN

## Abstract

Hepatocellular carcinoma (HCC) is one of the most lethal human cancers worldwide because of its high incidence, its metastatic potential and the low efficacy of conventional treatment. Inactivation of apoptosis is implicated in tumour progression and chemotherapy resistance, and has been linked to the presence of endoplasmic reticulum stress. Melatonin, the main product of the pineal gland, exerts anti-proliferative, pro-apoptotic and anti-angiogenic effects in HCC cells, but these effects still need to be confirmed in animal models. Male Wistar rats in treatment groups received diethylnitrosamine (DEN) 50 mg/kg intraperitoneally twice/once a week for 18 weeks. Melatonin was given in drinking water at 1 mg/kg/d, beginning 5 or 12 weeks after the start of DEN administration. Melatonin improved survival rates and successfully attenuated liver injury, as shown by histopathology, decreased levels of serum transaminases and reduced expression of placental glutathione S-transferase. Furthermore, melatonin treatment resulted in a significant increase of caspase 3, 8 and 9 activities, polyadenosine diphosphate (ADP) ribose polymerase (PARP) cleavage, and Bcl-associated X protein (Bax)/Bcl-2 ratio. Cytochrome c, p53 and Fas-L protein concentration were also significantly enhanced by melatonin. Melatonin induced an increased expression of activating transcription factor 6 (ATF6), C/EBP-homologous protein (CHOP) and immunoglobulin heavy chain-binding protein (BiP), while cyclooxygenase (COX)-2 expression decreased. Data obtained suggest that induction of apoptosis and ER stress contribute to the beneficial effects of melatonin in rats with DEN-induced HCC.

## Introduction

Hepatocellular carcinoma (HCC) is the fifth most common disease in men worldwide, the seventh in women and the third leading cause of cancer-related mortality. It has been established as the final step for chronic liver diseases, and it is closely related to fibrosis and cirrhosis with a variable aetiology [1]. Its incidence and mortality are higher in developing regions, but most of the burden occurs in developed countries [2]. In developed countries (North America, Europe and Japan) infection with hepatitis C virus (HCV) and alcohol use are the main risk factors. However, in underdeveloped countries HCC is associated with in hepatitis B (HBV) and exposure to aflatoxin B1 [3]. The late diagnosis and the low efficacy of drugs employed in its treatment make HCC the third cause of cancer death. Moreover, it is well known that HCC develops resistance to chemotherapeutic agents, which complicates HCC management [1].

Resistance to cell death is a distinctive characteristic of cancer. Apoptosis is one of the main mechanism implicated in cell death, and its inactivation contributes to tumour progression and chemotherapy resistance [4]. Apoptosis can be triggered by different pathways that depend on the initial stimulus. The extrinsic pathway requires the activation of transmembrane receptors by soluble death ligands, such as Fas ligand (Fas-L), that initiate a cascade of events which finally stimulate caspase 3 pro-death activity [5]. The intrinsic pathway involves the mitochondrial release of cytochrome c, allowing apoptosome complex formation and consequently procaspases activation. The permeabilization of mitochondrial membrane is necessary for the translocation of cytochrome c to cytosol, and this process is regulated by the Bcl-2 family of proteins [6]. The cascade of apoptosis signal transduction begins by the action of initiator caspases that are recruited and activated by autocatalytic processing. Among these caspases, caspase 8 and 9 are the main initiators of programmed cell death; caspase 8 is stimulated in response to extrinsic death ligands, while caspase 9 is necessary for the activation of executor caspase 3 [7]. Targeting both the intrinsic and extrinsic pathways reduces the growth of different tumour types, and a large number of studies demonstrate that different drugs alone or in combination enhance apoptosis in cancerous cells, including HCC [8].

Various cell stress factors lead to the accumulation of unfolded proteins in the endoplasmic reticulum (ER), causing an imbalance that activates the unfolded protein response (UPR) in an attempt to ameliorate the protein accumulation in ER. Different pathways which are initiated by sensor proteins located in the membrane of the ER have been found as mediators of the ER stress response. Under non-extreme conditions, these proteins are inactivated due to the immunoglobulin heavy chain-binding protein (BiP) to their luminal domains; however, when unfolded proteins are accumulated, BiP is sequestered, letting ER stress sensors to initiate the protective response. Even so, if damage is not improved, ER stress turns out lethal. ER stress is also deregulated in different pathologies, including cancer [9]. Due to the tumour microenvironment, ER stress is activated as an adaptive mechanism in certain types of cancer, such as breast, prostate or liver cancers [10,11]. However, apoptosis derived from non-resolved ER stress has also been observed, indicating that a sustained ER stress response could contribute to tumour cell death [12]. C/EBP homologous protein (CHOP) has been indicated as a crucial element for ER stress-induced apoptosis [13]. Management of ER stress could have anticancer properties in HCC cell lines, in which it has been reported that ER stress induction via PERK phosphorylation leads to an increase in cell death through CHOP activity [14].

Melatonin is an indole hormone that regulates important physiological processes in eukaryotic and prokaryotic organisms. Beneficial effects on a wide range of pathological situations strengthened its possible antitumor effect, and many studies have demonstrated that melatonin is able to prevent promotion and progression in different cancers [15]. Melatonin combined with immunomodulating therapy in patients with HCC that are not suitable for conventional therapy delays tumour progression and reverses tumour formation [16]. Proapoptotic effects of melatonin [17–21], and effects in combination with ER stress inducers [22,23] have been reported in different HCC cell lines. Interestingly, melatonin has been shown to induce apoptosis under ER stress situations in hepatoma cells via induction of CHOP and suppression of cyclooxygenase 2 (COX-2) [22]. However, in spite of the number of *in vitro* studies on the beneficial effects of melatonin, only results demonstrating effects on cancer development and oxidative stress are available in animal models of HCC [24–26], and underlying mechanisms of melatonin effects are not completely understood. This research was aimed to extend our previous in *in vitro* findings, which demonstrate cell cycle arrest and apoptosis induction in HepG2 cells through intrinsic and extrinsic pathway [17–20]. Given the relationship between apoptosis and ER stress, we decided to investigate the possible modulation of both processes by melatonin an *in vivo* diethylnitrosamine (DEN)-induced rat model of HCC.

## Material and Methods

### Animals and experimental design

Male Wistar rats weighing 140–150 g were used for this study and were obtained from the Central Animal Laboratory of the Federal University of Pelotas, Brazil. The rats were caged individually at 24°C, with a 12h light-dark cycle and free access to food and water until the time of experiments in the Animal Experimentation Division of the Hospital de Clinicas de Porto Alegre, Brazil. All experiments were performed in accordance with the Guiding Principles for Research Involving Animals (NAS) under protocol number 140311 and were approved by the Ethics Committee of the Hospital de Clinicas de Porto Alegre (Brazil).

To induce hepatocarcinogenesis animals were given DEN (Sigma Aldrich, St. Louis, MO) at a dose of 50 mg/kg body weight i.p. twice a week for the first three weeks and once a week from weeks 4 to 6 and 11 to 13 up to 19 weeks. A single dose of 2-acetylaminofluorene (2-AAF, 100 mg/ kg, Sigma Aldrich, St. Louis, MO) was administered in week 4 [27]. The animals were divided into five groups (n = 12): Control (CO); Control+melatonin (CO+MEL); DEN; DEN+melatonin from the 5th to the 19th week (DEN+MEL5) and (V) DEN+melatonin from the 12th to the 19th week (DEN+MEL12). Melatonin (Sigma Chemical Company, St. Louis, MO) was prepared three times a week by dissolving the drug (16 mg) in ethanol 250μL, 100%, vol/vol) [28]. This solution was then diluted with drinking water to a final concentration of 20 mg/L. Melatonin was administered daily for 90 days (group DEN+MEL5) and 45 days (group DEN+MEL12). In the CO+MEL group, 6 animals received melatonin for 45 days and 6 animals for 90 days; data were pooled because no differences were observed in the different tested parameters. Water bottles were covered with aluminium foil. Rats drank about 25 mL/day, and the average daily intake of melatonin was estimated to be 1 mg/kg/day, which was expected to rise 20–30 times normal plasma melatonin levels. Appropriate dilutions were made to the drinking melatonin solution to match individual variations in water consumption on the previous day [28].

After fasting for 12 hours, the animals were anesthetized with ketamine hydrochloride (Ketalar® 100 mg/kg) and xylazine (50 mg/kg) and subjected to blood collection for measurement of biochemical analysis. Liver samples were removed for histological analysis and comet assay. Samples of livers for histology, biochemical and molecular analyses were taken from the right medial lobe. The animals were killed at the end of the experiment by exsanguination under deep anesthesia, as described in the American Veterinary Medical Association (AVMA, 2013) Guidelines on Euthanasia.

### Biochemical analysis

Serum levels of alanine aminotransferase (ALT), aspartate aminotransferase (AST), gamma-glutamyl transferase (GGT), and alkaline phosphatase (AP) were measured with automated routine laboratory methods at Hospital de Clínicas de Porto Alegre, Brazil.

### Histology

For histological examination, a specimen of liver was trimmed and fixed by immersion in 10% buffered formalin for 24 hours. The blocks were dehydrated in a graded ethanol series and embedded in paraffin wax. Serial 3-μm sections were stained with hematoxylin and eosin (H&E) and imaged (200x magnification). Histological evaluation was performed by a highly qualified examiner in a blinded manner, assuring the reliability of the data.

### Immunohistochemical staining

Immunohistochemistry was used for detection of caspase 3 positive cells in liver samples. Tissue samples were fixed in 10% buffered formalin and embedded in paraffin. Sections of 4-μm were dewax and hydrated through a gradient of ethanol. Antigen retrieval was performed in 10 mM citrate, pH 6.0, by heating in a microwave oven for 25 minutes at 720 W. After cool down (30 minutes at room temperature), endogenous peroxidase was blocked by exposure to 3% hydrogen peroxide in methanol for 15 minutes. The slides were incubated with antibody cleaved-caspase 3 (1:50 dilution, Cell Signaling, MA) overnight at 4°C. After washing with PBS, slides were incubated with secondary Dako EnVision+System-HRP labelled polymer anti-rabbit (Dako North America, CA) and bound antibody was visualized by use of 3,3’-diaminobenzidine (DAB) substrate kit (Vector Laboratories, Burlingame, CA). Cell nuclei were counterstained with hematoxylin. The specificity of the technique was evaluated by negative controls.

### Comet assay

The alkaline comet assay was carried out as described by Tice et al [29] with minor modifications. Images of 100 randomly selected cells (50 cells from each of two replicate slides) were analysed from each animal. Cells were also visually scored according to tail size into five classes ranging from undamaged (0) to maximally damaged (4), resulting in a single DNA damage score for each animal and consequently for each studied group. Therefore, the damage index (Dl) can range from 0 (completely undamaged, 100 cells x 0) to 400 (with maximum damage, 100 cells x 4) and damage frequency (DF) was calculated based on the number of cell with tail versus those with no tail.

### Caspase-3, 8 and 9 activities assays

Enzymatic activities were performed in total extracts prepared by liver tissue homogenization in 0.25 M sucrose, 10 mM Tris, 1mM EDTA and a phosphatase and protease inhibitor cocktail (Roche Diagnostic) [30]. Protein concentration was determinated by Bradford assay. Equal amounts of protein were incubated for 60 min at 37°C in a buffer 20 mM HEPES, glycerol 10%, DTT 2 mM, pH = 7.4 containing 100 μM concentration of the specific fluorogenic Ac-DEVD-AMC, Ac-IETD-AFC and Ac-LEHD-AMC for caspase-3, -8 and -9 respectively (Alexis Biochemicals, Cornerstone, San Diego, CA, USA). Cleavage of fluorescent substrates was monitored using fluorescence microtitre plate reader (Synergy HT Multi-Mode Microplate Reader, Bio-Tek Instruments, Inc., Winooski, VT) at excitation/emission wavelengths of 360/460 nm for caspase-3 and -9, and 400/505 nm for caspase-8. Activity was expressed as fluorescents units per min of incubation.

### Western blot analysis

Western blot analyses were performed on cytosolic extracts prepared by liver tissue homogenization in 0.25 mM sucrose, 1 mM EDTA, 10 mM Tris and a phosphatase and protease inhibitor cocktail (Roche Diagnostic GmbH) [31]. The homogenate was incubated on ice for 30 min, and finally, the samples were centrifuged at 12,000 *g* for 30 min at 4°C. The supernatant fraction was recollected and stored at -80°C in aliquots until use. Protein concentration was measured by the Bradford assay. Equal amounts of protein (15–30 μg) were separated by 9–12% sodium dodecyl sulfate (SDS) polyacrylamide gel electrophoresis for 1.5 hr at 100 V and then blotted on polyvinylidene fluoride membranes (Amersham Pharmacia; Little Chalfont, UK). The membranes were then blocked with 5% non-fat dry milk in phosphate buffered saline buffer containing 0.05% Tween 20 (PBST) for 1 hr at room temperature and probed overnight at 4°C with polyclonal anti-PARP cleaved, Bcl-2, Bax, Fas-L, p53, COX-2, BiP, CHOP and ATF6 antibodies (Santa Cruz Biotechnology; Santa Cruz, CA) and GST-P polyclonal antibody (Enzo Life Science, Farmingdale, NY, USA) at 1:200–1:1,000 dilution with PBS-T containing 2.5% non-fat dry milk. Equal loading of protein was demonstrated by probing the membranes with a rabbit anti-β-actin polyclonal antibody (1:2,000 Sigma; St Louis, MO). After washing with TBST, the membranes were incubated for 1 hr at room temperature with secondary HRP conjugated antibody (Dako, Glostrup, Denmark, 1:4,000) and visualized using ECL detection kit (Amersham Pharmacia; Uppsala, Sweden) [32]. The density of the specific bands was quantified with an imaging densitometer (Scion Image; Maryland, MA).

### Statistical analyses

Results were expressed as mean values. Significant differences between means were evaluated by one-way analysis of variance; in the case of significance, the means were compared with the Bonferroni test. Significance was accepted when *P* value was less than 0.05. Values were analyzed using the statistical package SPSS 22.0 (IBM Corporation, Armonk, NY).

## Results

Serum biochemical analyses were performed to determine hepatic function. Both ALT and AST activities showed a significant increase in DEN-treated group which was significantly prevented by melatonin. AP and GGT also exhibited an increase in DEN group that was partially inhibited by melatonin. The hepatic-somatic ratio was significantly higher in DEN animals than in melatonin-co administered groups (Table 1).

**Table 1:** Effects of DEN and melatonin on serum enzyme levels and hepatosomatic ratio (HSR).

	Group				
Marker	CO	CO+MEL	DEN	DEN+MEL5	DEN+MEL12
AST (U/L)	132 ± 15.5	107 ± 7.7	235 ± 71*	166 ± 59* ^#^	143 ± 28* ^#^
ALT (U/L)	79 ± 6	54 ± 15	124 ± 39*	102 ± 37* ^#^	96 ± 19* ^#^
AP (U(L)	182 ± 42	111 ± 50	316 ± 99*	215 ± 98* ^#^	213 ± 46* ^#^
GGT (U/L)	7.8 ± 2.5	9.0 ± 2.1	50.3 ± 9.0*	35.8 ± 11.0* ^#^	25.1 ± 9.0* ^#^
HSR (%)	3.1 ± 0.2	2.9 ± 0.3	10.8 ± 5.0*	4.6 ± 1.2^#^	4.2 ± 0.7^#^

*AST*, aspartate aminotransferase; *ALT*, alanine aminotransferase; *AP*, alkaline phosphatase; *GGT*, gamma-glutamyl transferase; *HSP*, hepatosomatic ratio.

Values are expressed as means ± standard error of the mean.

*p<0.05, compared with control (CO).

^#^p<0.05 compared with DEN.

**Fig 1:**
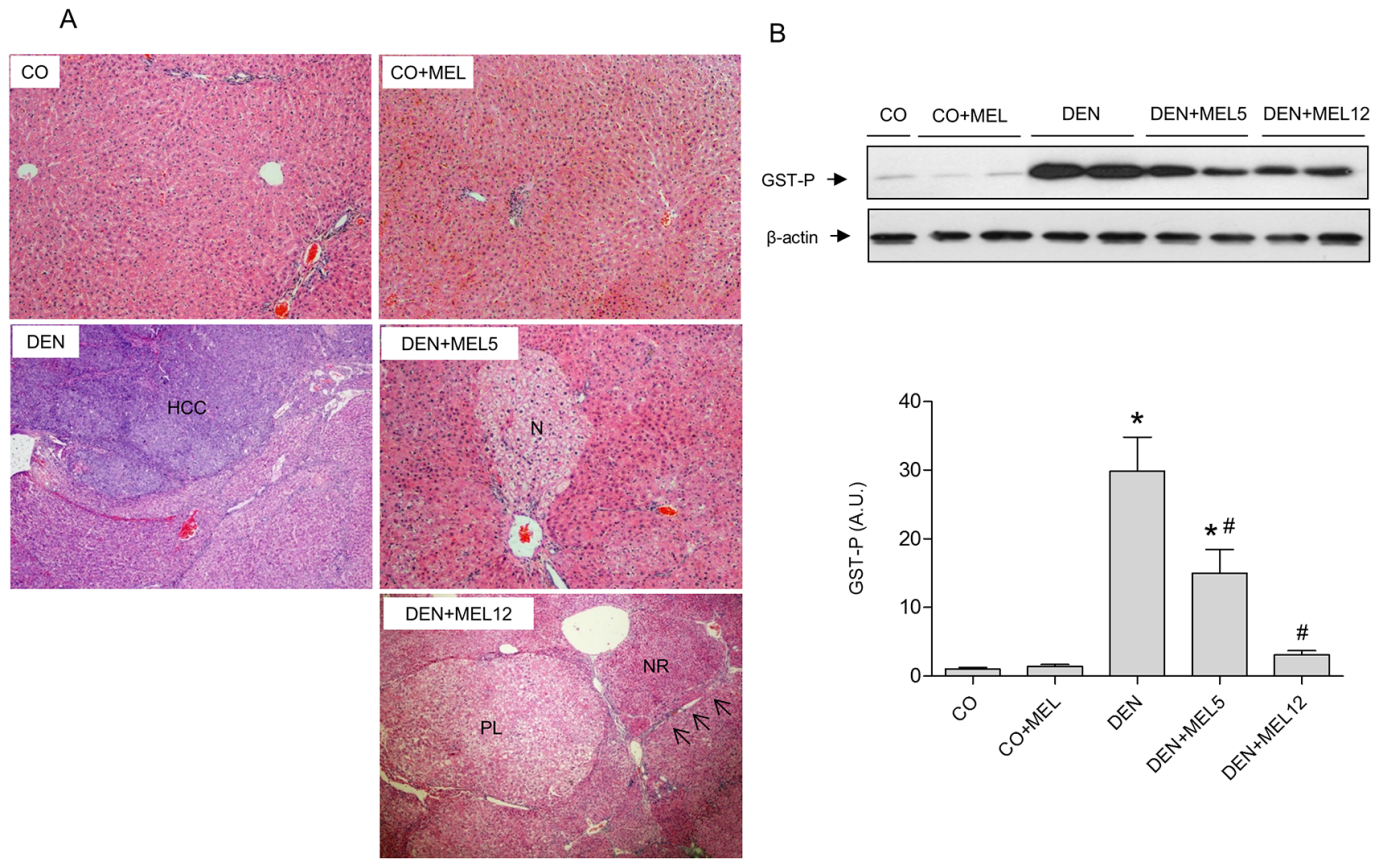
Effect of DEN and treatment with melatonin on HCC development. (A) Histologic assessment of liver sections from control (CO), control rats treated with melatonin (CO+MEL), DEN-treated rats (DEN), and DEN and melatonin treated rats beginning 5 or 12 weeks after the start of DEN administration (DEN+MEL5 and DEN+MEL12, respectively). The livers of CO and CO+MEL had normal architecture, and DEN treatment induced advanced HCC. Treatment with melatonin resulted in a significant amelioration in histologic changes; DEN+MEL5 showed only some dysplastic foci (N), and DEN+MEL12 multiple nodules of regeneration (NR), precancerous lesions (PL) with fibrosis and cirrhotic pattern (arrows). (B) Representative Western blot and densitometric quantification of GST-P protein levels. Equal loading of protein was assessed by β-actin analyses. Values are expressed as means ± standard error of the mean.*p<0.05, compared with control (CO), ^#^p<0.05, compared with DEN.

Microscopy observations revealed that DEN induced chronic damage and lymphocytic infiltration. Livers of DEN-treated rats presented areas characterized by large nucleolus and increased mitotic index, and hepatic parenchyma was altered, with pseudotrabecular and pseudoacinar growth pattern showing large HCC nodules. Treatment with melatonin resulted in a significant amelioration in histologic changes; DEN+MEL5 showed only some dysplastic foci, and DEN+MEL12 samples showed multiple nodules of regeneration, precancerous lesions with fibrosis and cirrhotic pattern (Fig 1B).

Placental GST-P is expressed during hepatocarcinogenesis in rats, and is used as a HCC marker. GST-P protein levels were analyzed by Western blot, showing an increase in DEN group. However, in melatonin plus DEN-treated animals, its expression was significantly lower (Fig 1C), reaching a higher extent in the late-stage treated group. The study of DNA integrity was assessed by comet assay in blood and liver. No significant alterations were observed in blood, but there was a significant increase in damage index in DEN group compared to control in hepatic tissue. Moreover, melatonin supplementation reduced DNA damage and damage frequency compared to DEN group (Table 2).

**Table 2:** Comet assay in the peripheral blood and liver from rats treated with DEN and melatonin.

Tissue	Group	Damage Index	Damage frequency
**Blood**	CO	7.6 ± 7.1	3.4 ± 3.4
	CO+MEL	7.0 ± 5.1	3.9 ± 4.4
	DEN	9.3 ± 5.1	6.3 ± 2.2
	DEN+MEL5	7.0 ± 2.4	6.4 ± 2.4
	DEN+MEL12	8.7 ± 3.3	7.7 ± 3.0
**Liver**	CO	95.8 ± 27.6	74.6 ± 17.9
	CO+MEL	96.5 ± 17.6	79.5 ± 16.9
	DEN	339.3 ± 56.5*	99.0 ± 2.0*
	DEN+MEL5	259.0 ± 42.8* ^#^	92.3 ± 5.6
	DEN+MEL12	119.6 ± 17.9 * ^#^ ^&^	60.7 ± 5.0* ^#^ ^&^

Damage index can range from 0 (completely undamaged) to 400 (with maximum damage); damage frequency was calculated based on the number of cells with tail versus those with no tail.

Values are expressed as means + standard error of the mean.

*p<0.05, compared with control (CO).

^#^ *p*<0.05 compared with DEN.

^&^p<0.05 compared with DEN+MEL5.

**Fig 2:**
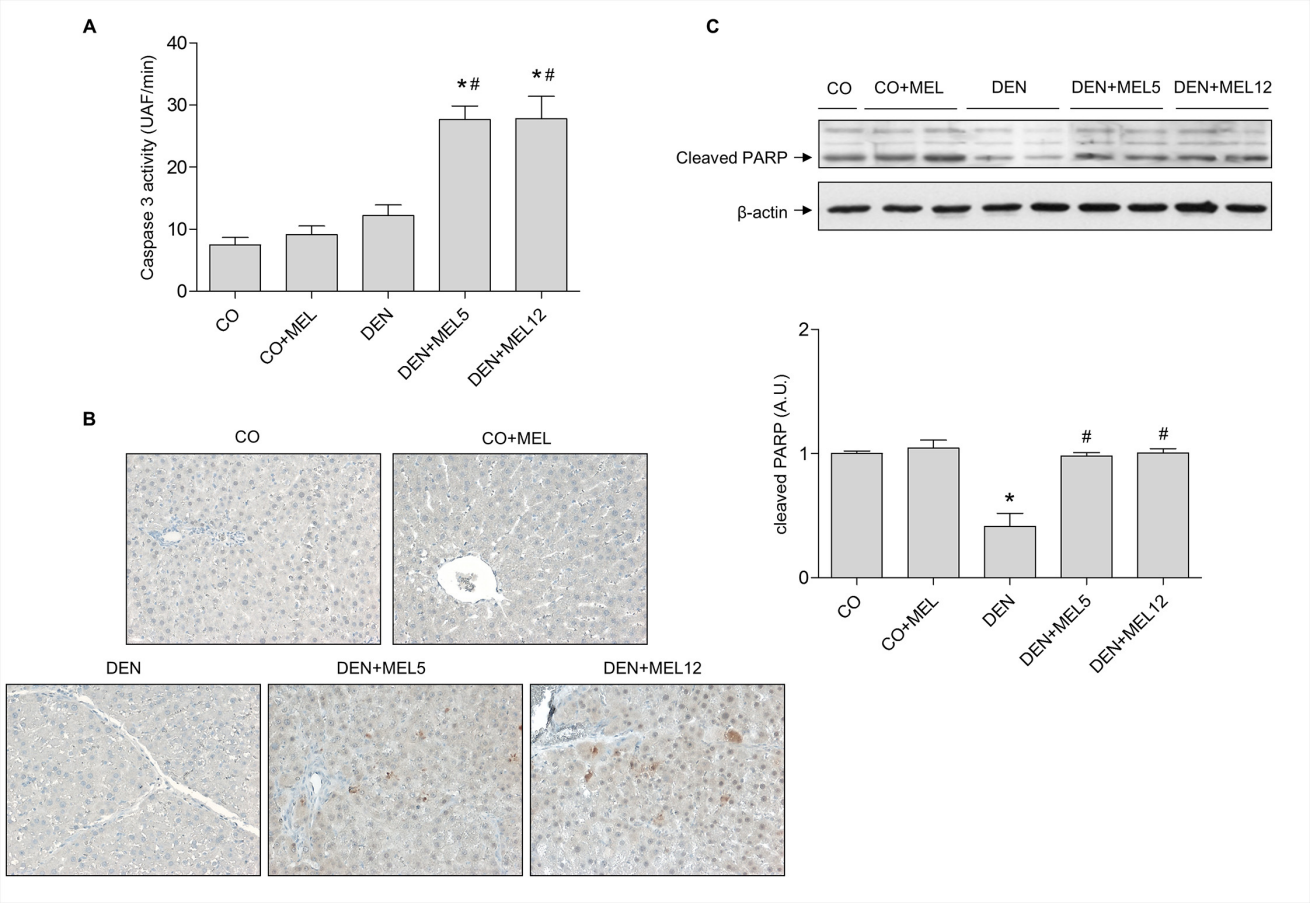
Effect of DEN and treatment with melatonin on markers of apoptosis. (A) caspase 3 activity was analysed with a fluorescent analogue of caspase 3 substrate and fluorescence measurement. (B) Histologic assessment of activated caspase 3 showed a higher expression in DEN plus melatonin groups compared to controls (200x). (C) Representative Western blot and densitometric quantification of PARP cleavage. Equal loading of proteins was assessed by β-actin analyses. Values are expressed as means ± standard error of the mean.*p<0.05, compared with control (CO) ^#^p<0.05, compared with DEN.

To elucidate the possible proapoptotic effect of melatonin, we performed several analyses on the apoptotic pathway. The status of PARP and caspase-3 was evaluated as final markers of apoptosis. We observed that cleaved PARP expression decreased in DEN-treated animals and was significantly augmented by melatonin, showing a restoration of normal levels (Fig 2C). Caspase 3 activity was measured by incubating samples with a specific fluoregenic substrate. An increase in fluorescence was observed when melatonin was supplemented in DEN-treated animals (Fig 2A). Moreover, active caspase 3 positive foci were observed by immunohistochemistry in livers of DEN plus melatonin groups compared to control animals (Fig 2B). Activation of the executor caspase 3 indicates that melatonin is able to induce apoptosis, suggesting that death of cancer liver cells could contribute to the improvement in liver histology.

**Fig 3:**
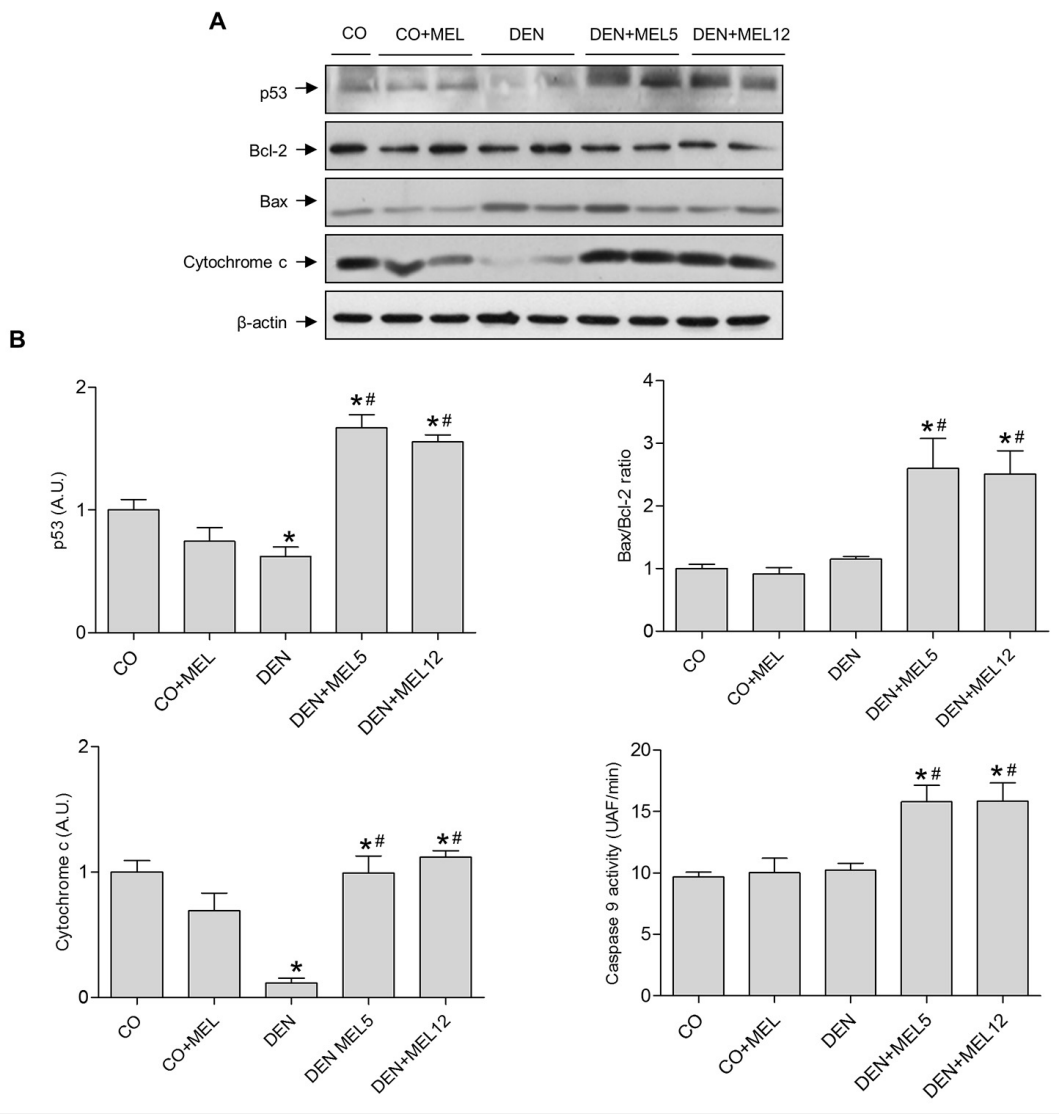
Effect of DEN and treatment with melatonin on the intrinsic pathway of apoptosis. (A) Representative Western blot of intrinsic apoptotic proteins p53, bcl-2, Bax and cytochrome c in the cytosolic fraction, and caspase 9 activity. Equal loading of proteins was assessed by β-actin analyses. (B) Densitometric quantification of Western blots. Values are expressed as means ± standard error of the mean.*p<0.05, compared with control (CO), ^#^p<0.05, compared with DEN.

**Fig 4:**
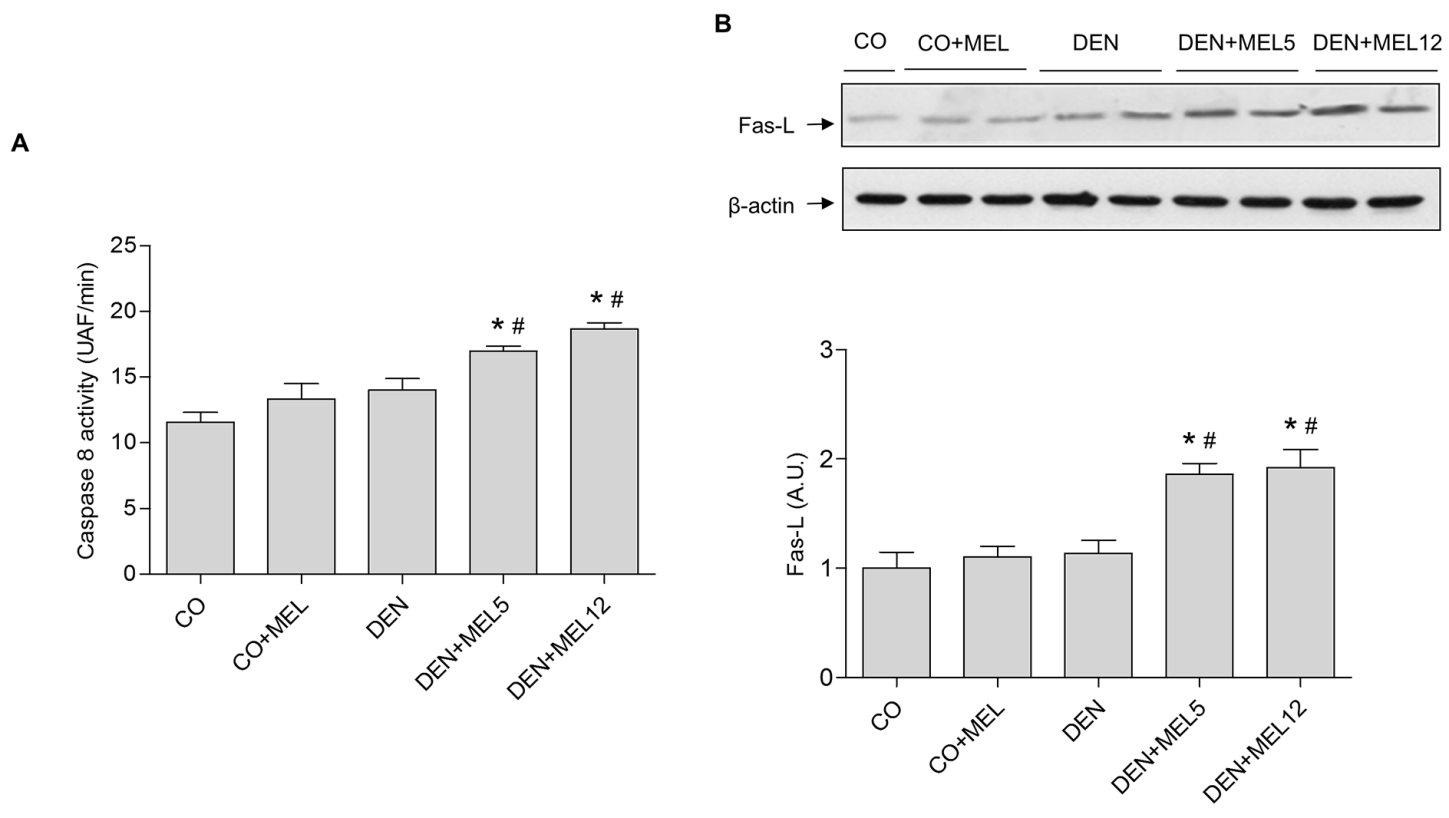
Effect of DEN and treatment with melatonin on the extrinsic pathway of apoptosis. (A) caspase 8 activity was analysed with a fluorescent analogue of caspase 8 substrate and fluorescence measurement. (B) Representative Western blot and densitometric quantification of Fas-L. Equal loading of protein was assessed by β-actin analyses. Values are expressed as means ± standard error of the mean.*p<0.05, compared with control (CO), ^#^p<0.05, compared with DEN.

To clarify the mechanism of apoptosis induced by melatonin, markers of the intrinsic apoptotic pathway were analyzed by Western blot. Caspase 9 activity showed an increase in melatonin groups compared to control and DEN animals. The balance between apoptotic and anti-apoptotic proteins was assessed by the Bax/bcl-2 ratio. Although DEN was not able to alter this relation, melatonin administration increased the Bax/bcl-2 ratio (Fig 3). Moreover, mitochondrial dysfunction was evaluated by measuring cytochrome c protein levels in cytosolic liver fractions; cytochrome c expression decreased significantly in DEN group and was significantly increased in melatonin-supplemented animals (Fig 3). p53 expression was also significantly reduce in DEN rats and increased in those receiving melatonin (Fig 3). All these data suggest that melatonin is able to increase intrinsic apoptosis in HCC. Because of apoptosis can also be managed by an extrinsic pathway, we evaluated the expression of Fas-L, and caspase 8 activity. As shown in Fig 4, both caspase 8 activity and Fas-L protein levels were not altered by DEN, but augmented when melatonin was administrated to DEN-treated animals. These observations suggest that not only intrinsic but also extrinsic apoptosis is induced by melatonin in HCC.

**Fig 5:**
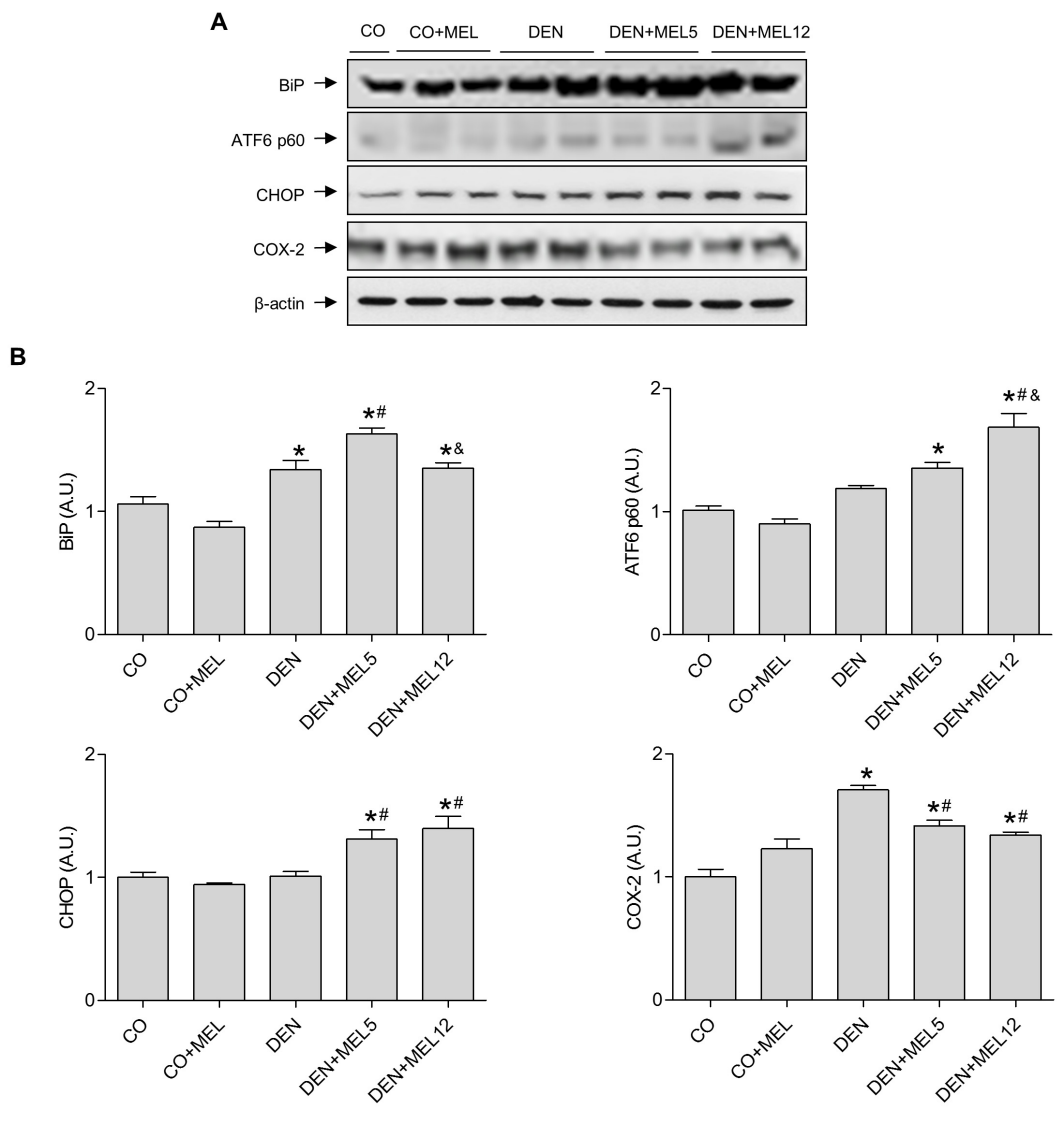
Effect of DEN and treatment with melatonin on markers of ER stress and COX-2. (A) Representative Western blot of BiP, ATF6 p60, CHOP and COX-2. Equal loading of proteins was assessed by β-actin analyses. (B) Densitometric quantification of Western blots. Values are expressed as means ± standard error of the mean.*p<0.05, compared with control (CO), ^#^p<0.05, compared with DEN, ^&^p<0.05 compared with DEN+MEL5.

ER stress is one of the mechanisms altered during hepatocarcinogenesis and is closely related to apoptosis, so we decided to evaluate the status of ER markers. Melatonin addition induced CHOP and BiP expression in both melatonin-treated groups, (Fig 5). ATF6 is one of the sensor proteins which, after cleavage, activate the expression of CHOP and BiP. ATF6 proteolysis was analysed by Western blot, showing that melatonin was able to increase the levels of the active form ATF6 p50 compared to DEN and control groups (Fig 5). This induction was larger in the group of rats that received melatonin at an advanced stage. These observations indicate that the indole is able to induce ER stress in liver animals with HCC. COX-2 has been shown to regulate both, apoptosis and ER stress. To define the possible implication of COX-2 in the effects of melatonin action, we analyzed COX-2 protein levels, and observed that DEN increased the expression of this protein, but melatonin supplementation reduced significantly its levels (Fig 5), suggesting a possible role of COX-2 in the apoptotic and ER alteration effects of melatonin in HCC.

## Discussion

Among the different *in vivo* models that have been developed to induce HCC, the DEN model has been widely used. Here, we have used a previously validated model of exposure to DEN [27] to address the effects of melatonin on HCC. Melatonin is known to present oncostatic properties by enhancing antioxidant systems and immune mechanisms, and modulating pathways that control cell cycle and cell growth [33–35]. However, mechanisms contributing to the beneficial effects of melatonin in HCC *in vivo* are not fully understood, so we decided to focus on the role of melatonin in the regulation of different molecular pathways related to cell death in HCC.

Administration of melatonin to DEN-treated animals indicated that the indole was able to reduce hepatocarcinogenic features in HCC-bearing rats, because it ameliorated histology, and reduced changes in serum enzymes and in the expression of GST-P, a protein considered a marker of hepatocarcinogenesis[36]. These findings are in accordance with results by different authors that also used DEN as a HCC promoter and tested effects of melatonin or other antioxidants, such as resveratrol, maotai or daffron, in rats [24,37–40] and mice [41]. Melatonin also prevented DNA damage in DEN-treated animasl, probably due to its capacity to regulate several key genes involved in DNA repairing pathways, such as CEP152 and N4BP2L2 [42]. However, other natural compounds with hepatoprotective properties such as silymarin have not demonstrated an anticarcinogenic effect in this model of HCC [37], although this molecule has been found to be able to inhibit tumour growth *in vitro* [43]. Experimental designs, doses utilized and time of the experiments could contribute to explain different effects of these compounds in animal and *in vitro* models.

Anti-oncogenic properties of melatonin have been carried out in cultured hepatoma cells, where melatonin modulates apoptosis, inhibits the expression of angiogenic genes and reduces the migration and motility of HepG2 cells during metastasis, with no toxic effects on healthy hepatocytes [17–21]. However, the effects of melatonin and other natural compounds in the DEN model are mainly focused on their impact on antioxidant machinery but not in the molecular mechanisms that have been observed *in vitro*, so it is necessary to confirm the observations made in culture cells to define effective targets for HCC treatment [24,26,44–46].

Because apoptosis disturbances are key events in cancer we decided to evaluate the effect of melatonin on the apoptotic status in DEN-treated rats. Caspase 3 is the main executor of apoptosis and its activation results in PARP proteolysis [47]. Our data indicate that melatonin increased PARP cleavage and caspase 3 activity, confirming its proapoptotic effect. These results are in agreement with our previous findings in HepG2 cells [17]. Combination of melatonin with chemotherapeutics such as doxorubicin or sorafenib [48,49] also increase apoptosis in hepatoma cells. In addition, other antioxidants have also demonstrated pro-apoptotic properties in HCC induced by DEN injection [38,39,50,51], indicating that beneficial effects of these natural compounds in HCC are possible linked to apoptosis induction.

To clarify the proapoptotic mechanism of melatonin, and based on our previous studies *in vitro*, we decided to evaluate the status of the intrinsic and extrinsic apoptotic pathways. Intrinsic pathway is frequently aberrant in cancer, and Bcl-2 protein family members are expressed abnormally in cancer cell lines and tumour human samples [52]. We observed that Bax protein levels increased in animals with HCC that received melatonin. It is known that Bax is inactivated in solid tumours [53], so restoration of its functionality could lead to HCC regression. Bcl-2, the main anti-apoptotic protein is able to inhibit Bax action, and it has become in an important target for the development of new drugs [54,55], so the relationship between both proteins was analyzed. The Bax/bcl-2 ratio was higher in melatonin supplemented groups compared to DEN-treated animals. This effect correlated with the release of cytochrome c from the mitochondria, indicating that regulation of the mitochondrial pathway contributes to melatonin effects, as it has been observed in HepG2 cells [17]. Other antioxidants, such as resveratrol and morin have also been reported to increase Bax protein levels [38,51]. p53 acts as a transcription factor that regulates the expression of genes involved in apoptosis, such as Bax or bcl-2 [56]. Here, we have found that melatonin plus DEN-treated rats overexpressed p53 in hepatic tissue, suggesting that this protein could be involved in the regulation of intrinsic apoptotic markers by melatonin. Due to the fact that melatonin also activates the extrinsic pathway of apoptosis in HepG2 cells [17], we evaluated the activity of caspase 8, a key initiator caspase that mediates the death receptor pathway of apoptosis, in which the binding of Fas to Fas-L induces receptor clustering and formation of death-induced signalling complexes. Caspase 8 activity and Fas-L expression were also induced in HCC-bearing rats treated with melatonin. Melatonin also induces caspase 8 activation in H_2_O_2_-treated leukaemia HL-60 cells, but has no effect in leukemia Molt-3 cells [57,58], indicating that melatonin actions are cell type and context-dependent.

ER is an important organelle involved in protein folding, maintenance of calcium homeostasis and lipid biosynthesis. If cells are subdued to stressful situations, ER initiates a response to maintain cellular integrity (UPR), but in certain situations this response triggers cells to apoptosis [9]. Effects of compounds such as neferine or guggulsterone on the viability of hepatoma cells associate to ER induction [59–61]. Therefore, regulation of ER stress could also be involved in the pro-apoptotic actions of melatonin. We evaluated the effect of melatonin on protein levels of BiP, a chaperone responsible for the properly folding of proteins in the ER [62], and found an increased expression in DEN-treated rats that received melatonin, indicating that the hormone was able to initiate the ER stress response. ATF6 is as sensor protein cleavage when ER stress is activated, leading to the release of an active form that promotes the translation of chaperone genes and CHOP [63], a modulator of apoptosis that increases the expression of pro-apoptotic proteins and downregulates anti-apoptotic genes [13,64,65]. Our finding that melatonin increases the expression of cleaved ATF6 and CHOP in rats with HCC suggests that ER stress could be responsible, in part, for the enhancement of apoptosis in our model. Effects of melatonin on ER stress have been evaluated in HePG2 with the co-administration of an ER inductor, and it has been shown that melatonin treatment enhanced the levels of CHOP and decreased the Bcl-2/Bax ratio [22]. Moreover, it has been reported that melatonin strengthens the apoptotic effect of doxorubicin under ER stress conditions in HepG2 and SMMC-7721 hepatoma cell lines [23].

Since COX-2 inhibition has been shown to increase apoptosis in tunicamycin-treated HepG2 cells through induction of CHOP expression and downregulation of bcl-2/Bax ratio, it has been proposed to be a target molecule for HCC treatment [22]. Our results demonstrate that DEN treatment caused an increase in COX-2 protein levels while melatonin co-administration partially restored normal levels. COX-2 activity is inhibited by melatonin in HepG2 and SMMC-7721 cells, and blocking of AKT activation results in lowered COX-2 expression [66]. Therefore, suppression of the COX-2/PI3K/AKT pathway could contribute to induction of apoptosis through ER stress by melatonin. Other proteins such as forkhead box O3 (FOXO3) [19] or mitogen-activated protein kinases (MAPK) such as c-Jun N-terminal kinase (JNK) [17] could also play a role in melatonin-induced apoptosis in HepG2 cells. In this regard, JNK phosphorylation is altered during melatonin treatment in HepG2 cells [17], so it could be the link between ER stress and apoptosis since it has been demonstrated that its activation is induced under ER stress conditions and regulates apoptotic gene expression [67]. Therefore, more studies in the DEN model of HCC are necessary to confirm the findings obtained *in vitro*.

Curiously, our experiments suggest that melatonin treatment for the later 45 days could be more effective than a longer and earlier treatment, especially concerning GST-P level and nuclear damage. These apparently controversial results could be explained by the chronologic evolution of the DEN-induced hepatocarcinogenesis. Previous reports have demonstrated that HCC is stablished between 17–19 weeks of DEN administration, while inflammation starts after 4–5 weeks and cirrhosis appears 10–12 weeks after DEN injection [27]. Melatonin treatment was initiated in our study beginning 5 or 12 weeks after the start of DEN administration to assess effects when different degrees of liver damage were present. Although melatonin did not caused significant differences in most markers of apoptosis according to the time of administration, GST-P expression was notably lower when melatonin administration started during cirrhosis establishment. Effects of melatonin on HCC *in vivo* had not been studied until now. However, a previous research has analyzed resveratrol treatment, and it has been described that this antioxidant interferes with DEN-induced HCC at early and advanced stages, although in terms of caspase activation data obtained indicated a higher rate of conversion in the advanced stage, probably due to a higher demand to compensate DEN injury [38]. Differences among both studies could be related to the experimental design of DEN administration. However, we also observed that, although ER stress increased in the two groups of rats receiving DEN and melatonin, administration of the indole in the advanced-stage resulted in a higher capacity for ATF6 and CHOP induction, which suggests an increased/demand requirement for the induction of apoptosis. Data obtained indicate that melatonin could have different effects on molecular pathways depending on the degree of hepatic injury. Our results suggest that further studies are required to a better understanding of the melatonin mechanism against HCC and to confirm if its antitumor effect could be clinically relevant.

## Conclusions

Our research demonstrates that melatonin administrated during inflammation or cirrhosis progression acts as a powerful pro-apoptotic agent able to ameliorate histology and biochemical outcomes in DEN-induced HCC. Moreover, melatonin also induces an ER stress response that could be linked to the apoptotic effects observed. However, it is necessary to determine the signaling pathways that regulate both processes with the aim to potentiate the therapeutic applications of melatonin in HCC treatment.
